# Miniaturized, high numerical aperture confocal fluorescence detection enhanced with pyroelectric droplet accumulation for sub-attomole analyte diagnosis

**DOI:** 10.1364/BOE.504757

**Published:** 2023-11-03

**Authors:** Yunfeng Nie, Uusitalo Sanna, Teemu Sipola, Annukka Kokkonen, Inka Päkkilä, Juha Sumen, Katariina Rahkamaa-Tolonen, Volodymyr Tkachenko, Veronica Vespini, Sara Coppola, Pietro Ferraro, Simonetta Grilli, Heidi Ottevaere

**Affiliations:** 1 Vrije Universiteit Brussel and Flanders Make, Brussel Photonics, Dept. of Applied Physics and Photonics, Pleinlaan 2, 1050 Brussels, Belgium; 2 VTT Technical Research Centre of Finland Ltd, Kaitoväylä 1, FI-90571 Oulu, Finland; 3 Institute of Applied Sciences and Intelligent Systems, National Council of Research (CNR-ISASI), Via Campi Flegrei 34, 80078 Pozzuoli, Italy

## Abstract

To meet the growing demand for early fatal disease screening among large populations, current fluorescence detection instruments aiming at point-of-care diagnosis have the tendency to be low cost and high sensitivity, with a high potential for the analysis of low-volume, multiplex analytes with easy operation. In this work, we present the development of a miniaturized, high numerical aperture confocal fluorescence scanner for sub-micro-liter fluid diagnosis. It is enhanced with high-rate analyte accumulation using a pyroelectro-hydrodynamic dispensing system for generating tiny, stable sample droplets. The simplified confocal fluorescence scanner (numerical aperture 0.79, working distance 7.3 mm) uses merely off-the-shelf mass-production optical components. Experimental results show that it can achieve a high-sensitive, cost-efficient detection for sub-micro-liter, low-abundant (0.04 µL, 0.67 attomoles) fluid diagnosis, promising for point-of-care diagnosis.

## 1. Introduction

Currently, there is a growing demand for the early diagnosis of fatal diseases (Alzheimer diseases, cancers, etc.), which relies on the detection of multiple biomarkers in human body fluids at very low volume and low abundance (e.g., 1 µl and 0.1 femtomole) [1]. Thus, a robust, high-throughput, multiplexed, easy-to-operate screening approach to enable rapid detection of such analytes is highly motivated for point-of-care diagnosis. To identify molecules, two types of techniques are worth mentioning, label-free and labelling detection. Mass spectrometry is one of the representative label-free methods which is extremely sensitive [2], but it is also expensive, require specialized pre-treatment for sample preparation to eliminate disturbing particles [3], and cannot be used for fast screening. Optical label-free methods, such as surface plasmon resonance [4], waveguide plate [5] and photonic crystals [6,7], are attracting more attention for achieving extremely low limits-of-detection (LODs) down to aM. However, they are usually based on microfluidic chips which require hundreds of μl sample and show low potential for multiplexed detection.

The labelling techniques can enhance bioanalytical sensing with high specificity, including Enzyme-Linked Immunosorbent Assay (ELISA), chemiluminescence (CL), and fluorophores. ELISA and CL can detect low-abundant molecules down to 1 femtomole, but they typically require ∼100μl or more volume for one diagnosis [8–10]. Among them, fluorescent labels have been widely adopted for clinical diagnostics due to fast signal acquisition, high possibility for multiplexing, and straightforward labeling process [11,12], but the fluorescent signal is typically low in case of low-abundant analytes. Furthermore, metal nanostructures and plasmonic enhancement fluidic systems have promoted the detection sensitivity to unprecedented levels [13–15], but they also rely on hundreds of μl sample, and the highly sophisticated fabrication of their disposable sample reaction slides make them quite expensive for one detection. In contrast, confocal fluorescence has the advantages of using cost-effective disposable slides, easy preparation of samples and multiplex detection. Different strategies have been proposed to enhance the detection efficiency, including high numerical aperture (NA) objectives [16] and super critical angle fluorescence [17]. However, they have very shallow working distances and require a complicated opto-mechanical design to ensure high-performance.

This work focuses on a miniaturized, high NA confocal fluorescence scanning system that utilizes pyroelectric sample manipulation to allow low-volume, low-abundant molecule detection. The detected molecules (0.04 µL, 0.67 attomoles) in distilled water are much lower than state-of-the-art methods by using sample volumes around 100 µL to achieve a LOD of 0.1∼1pM [9,10,18,8], which equals to about 10∼100 attomoles. We also strive to measure a very challenging extremely low-abundant fluorescent sample in artificial urine with merely 0.04µl of 0.8pg/ml (∼0.21 zeptomoles). The pyroelectric sample dispensing can accumulate tiny droplets to increase the analyte concentration, with high position accuracy and constant spot geometry. On top of the generated highly concentrated, tiny-spot sample, the confocal fluorescence optical detection system is simplified with merely off-the-shelf optical components while maintaining a high NA and long working distance (WD). By a thorough investigation of the influential factors (selection of optics and filters, scattering on slides, etc.), we built the confocal fluorescence detection prototype (NA = 0.79, WD = 7.3 mm) to verify the proposed method. Experimental results on 40nl, 2.5 ng/ml antibody labelled fluorophores show an average signal-noise-ratio (SNR) of ∼100, successfully demonstrating a high potential for detecting low-abundant fluorescent analytes.

## 2. Materials and methods

### 2.1 Method overview

**Fig. 1.**
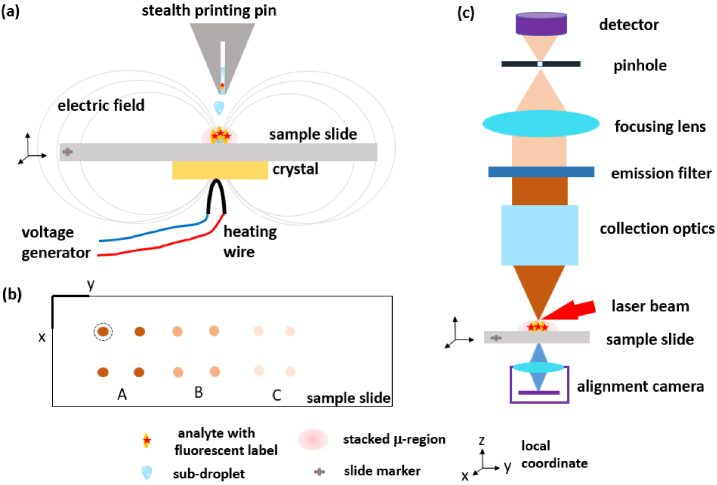
The detection method consists of two steps (a) the droplet-split-and-stack (DSS) dispensing using pyroelectric effect to generate constant and uniform sample spots and (c) the confocal fluorescence readout (CFR) to obtain the fluorescence signals, linked by (b) the sample slide with the same local coordinate. The dispensed spots contain different groups of prepared samples and blank controls. Since each of them has known coordinates from the DSS dispensing, the CFR only scans a small region (e.g., the dashed circle of group A) to accelerate the readout process. The alignment camera is used to find the marker and make sure the slide is well aligned.

The developed low-volume, low-abundant molecule detection method consists of two steps, the sample pyroelectric dispensing, and the confocal fluorescence readout (CFR), respectively as illustrated in Fig. 1(a) and (c). The dispensing step accumulates the sample in a droplet-split-and-stack (DSS) manner by pyroelectric effect, which reduces the droplet diameter to a constant, stacked micro-region and improves the concentration on the sample slide [19,20]. The sample droplet is prepared with a low concentration of analytes, usually conjugated with a certain fluorescent label, which could be a variety of analytes applicable to different diseases including cancers and cardiovascular diseases. The CFR module detects the fluorescence signals that are constraint to a micro-region with known local xy- coordinates from DSS dispensing, which reduces the confocal laser scanning to a smaller region (dashed circle in Fig. 1(b)) and eliminates the necessity to clearly “see” the spots in the collection path. These two factors enable the simplification of a high NA, long WD confocal optical microscopic system.

### 2.2 Pyroelectric sample accumulation

The DSS dispenser enables the sample manipulation by creating a pyro-electric field [21], which draws tiny sub-droplets from a micro-printing pin and stacks them onto a micro-region of the sample slide. A pyroelectric crystal (e.g., lithium niobate, LN) can generate a high electric field upon an appropriate variation of its temperature from the heating element.

A conventional Tungsten wire, around 300 µm thick, was bent appropriately to get a localized heat source in contact with the LN crystal, as shown in Fig. 1(a). The thermal stimulus was controlled by the power dissipation in the heating wire, which was driven by a voltage generator to generate electrical waveforms. We used a step function where the ‘on’ and ‘off’ times corresponded to the time lapse in which the current passed through the wire. It is well known that temperature values exceeding 37°C are detrimental for biomolecules. Therefore, we measured the temperature of the surface of the target slide by an infrared camera under different values of voltage and on/off times, to find the conditions that produce repeatable jets, but at the same time respect the limit of temperature. The optimal “on” and “off” times were 3.5 and 56.5 seconds, respectively. This allowed the slide temperature to vary between maximum and minimum values of 35°C and 26°C, respectively, with a tolerance of 0.1°C. The detailed implementation and spot quantification of the DSS dispensing unit can be found in our recent work [22].

To enable a rapid readout within a smaller region, the DSS dispenser can record the local coordinates of each spot. The spot locations are used for the CFR module to find the spots with a top-view CMOS alignment camera (IDS U3-3060CP-C-HQ). They are determined from one sample slide corner in the same orientation. Both the DSS and CRF modules are equipped with identical sample slide holders and XY translation stages (Zaber, X-LSM050A) to ensure an accurate reading of the spot locations.

### 2.3 Confocal fluorescence scanner

**Fig. 2.**
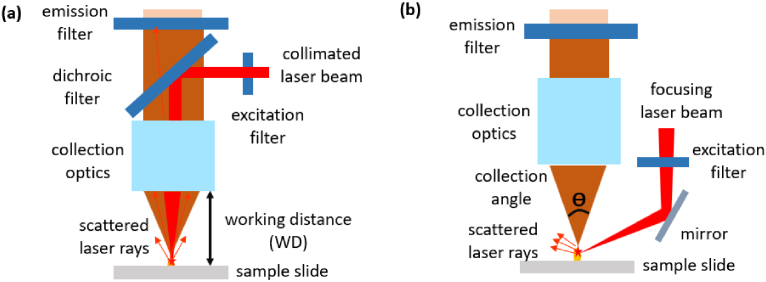
The confocal fluorescence readout (CFR) module has two different illumination strategies (a) reflected brightfield (perpendicular) illumination and (b) reflected dark-field (slanted) illumination which is more feasible with a long WD.

Confocal microscopy uses a spatial pinhole to block out the signal that is out of focus, ensuring a high SNR. A conventional confocal fluorescence scanner comprises of a laser beam, filters, collection optics, a pinhole, a focusing lens and a detector, as seen in Fig. 1(b). It is usually an expensive, desktop-size high-end instrument for versatile microscopic imaging tasks, such as the InnoScan 710 and the Typhoon image scanner. It is very typical to use brightfield or perpendicular illumination, as shown in Fig. 2(a), for most fluorescence microscopes, while an alternative darkfield or slanted illumination also exists as shown in Fig. 2(b). To collect the fluorescence signals efficiently, the excited sample spot must be conjugated with the pinhole, therefore the laser spot, the sample and the pinhole should be well aligned. The perpendicular-illumination path is easier for alignment, however, more laser light will be reflected to the collection path and become noise on the detector. The performance of these two configurations is therefore compared in Sec. 3.1.

Besides the illumination path, we consider two parameters for choosing the collection path, the achievable collection angle θ for a high throughput (NA=n.sin⁡(θ)) , n is the refractive index) and a long WD for point-of-care applications. From the perspective of obtaining higher throughput, different strategies of collection optics have been investigated [23,24], including gradient-index (GRIN) lenses [25], microscopic objectives [16] and off-the-shelf singlet lenses [26,27]. GRIN lenses are super compact, but they have a relatively low collection efficiency (e.g., NA ≤ 0.5). A microscope objective is certainly good for extended targets, but the high NA ≥ 0.6 and long WD ≥ 2 mm requirements imply a quite high cost. A pioneer attempt of using off-the-shelf singlet have been demonstrated in point scanning fluorescence microscopy with a high NA 0.85 and WD 0.36 mm [26]. Our proposed method uses the DSS dispenser to jet sample spots with a small, uniform size and known coordinates, such that the CFR module can be simplified to readout the fluorescence signals without the necessity to ‘see’ the spots. Therefore, we can use a single aspheric lens to achieve a high NA and long WD for the collection optics, and the optical performance is evaluated in terms of SNR in the following section.

### 2.4 Generalized SNR calculation

To evaluate the performance, we propose a modeling method to calculate the laser induced fluorescence signal and noise in such a confocal fluorescence detection path, and thus to approximately obtain the SNR as the criterion. This SNR performance can be used to optimize the CFR optics and select optimal components.

Firstly, the laser is propagated through illumination optics and focused on the fluorophore sample spot. Assume the laser power $P_{o}(\lambda )$ has reached the sample, the fluorophore absorb laser power based on Beer-Lambert law [28], as illustrated in Supplement 1. The total absorbed power is calculated as:

(1) $${\bar{P}}_{abs}=\eta_{L}.\eta_{N}\int P_{abs}(\lambda )d\lambda =\log(10).\text{DMAC}.c.d\int P_{0}(\lambda )\Phi_{A}(\lambda )d\lambda$$

Where, the fluorophore type determines its molar concentration c, absorption spectrum $\Phi_{A}$ , maximum decadic molar absorption coefficient (DMAC) and quantum efficiency $\eta_{\text{F}}$ , d is the sample thickness.

**Fig. 3.**
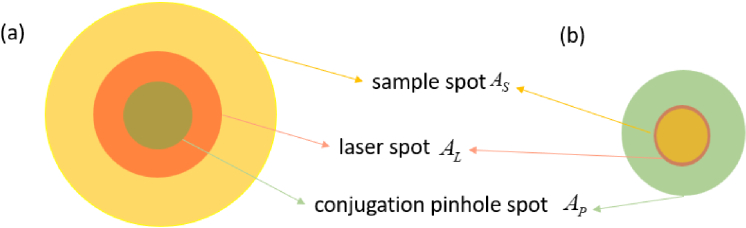
(a) An exemplary area relation of the sample spot area, laser spot and conjugation pinhole spot and (b) an alternative case with maximum absorbed laser and effective fluorophores $A_{L}\approx A_{S}\leq A_{P}$

Note that under confocal detection condition, the laser spot, the sample spot and the conjugation pinhole spot cannot be well overlapped due to imperfect component fabrication and alignment issues. Thus, in most cases, the laser power is not fully absorbed, and one confocal laser scan is not integrating the signal of all the fluorophores. We set the portion $\eta_{L}$ of laser can effectively illuminate the fluorophores, and the portion $\eta_{N}$ of the excited fluorescent light can reach the detector due to limited pinhole size, as seen in Fig. 3(a). Assuming the illumination is uniform and the power is not saturated, we can calculate the approximate absorbed laser percentage $\eta_{L}$ as the ratio of the sample spot area $A_{S}$ and the laser spot area $A_{L}$ , if $A_{L}\leq A_{S},\eta_{L}=1$ . Assume the fluorophores are uniformly distributed, the effective fluorophore percentage $\eta_{N}$ is calculated as the ratio of the conjugation pinhole spot area $A_{P}$ to the sample spot size $A_{S}$ , if $A_{P}\geq A_{S},\eta_{N}=1$ . Suppose the laser intensity is sufficient, if $A_{L}\approx A_{S}\leq A_{P}$ , both the absorbed laser and effective fluorophore percentages can be maximized, illustrated in Fig. 3(b). However, this case is practically difficult due to alignment issue and not easy to distinguish from dust “hotspot” noise.

In the emission optical path, as the fluorescence light is typically emitted in Lambertian distribution, the effective signal that reaches the detector is derived as:

(2) $$S=\int P_{em}(\lambda )d\lambda =\frac{\Omega }{4\pi }.\eta_{F}{\bar{P}}_{abs}\int \Phi_{E}(\lambda ).T_{em}(\lambda ).T_{opt2}(\lambda ).T_{Di}(\lambda ).\eta_{D}(\lambda )d_{\lambda }$$

Where, Ω is the solid angle related to the objective numerical aperture (NA), $\eta_{\text{F}}$ is the quantum yield of the sample fluorescence dye, $\eta_{\text{D}}$ is the quantum efficiency of the detector, $\Phi_{E}$ is the normalized fluorophore emission spectrum, $T_{em},T_{opt2},T_{Di}$ are respectively the transmission spectra of the emission filter, emission optical lenses and dichroic filter; if no dichroic filter is present, ${\text{T}}_{Di}\text{ = }1$ .

After the signal is known, we need to quantify the three main noises: laser scatter noise, autofluorescence and photodetector dark current noise, denoted by $N_{L},N_{AF},N_{dc}$ respectively. Autofluorescence is not considered in this work. The scatter noise is the excitation laser in the form of unblocked stray light, mainly due to scattering on the sample plane, which is quantitively obtained by the scatter yield $\eta_{sct}$ described in our recent work [29].

(3) $$N_{L}\text{ = }\eta_{sct}(\Omega )\int P_{0}(\lambda ).\Phi_{E}(\lambda ).T_{em}(\lambda ).T_{opt2}(\lambda ).T_{Di}(\lambda ).\eta_{D}(\lambda )d\lambda$$

When using a PMT or other photodetectors, the dark current noise can be obtained from the sensor specifications. In a confocal configuration, most of the autofluorescence will be excluded by the tiny pinhole close to the detector, thus this noise is negligible.

The SNR is calculated by

(4) $$\text{SNR = }\frac{S}{N_{L}+N_{dc}}$$

The modeling program is written in MATLAB, and can quickly give the estimated SNR once the related data specifications are obtained. The data related to laser and optical components can be either obtained from the providers or measurements, while the fluorophore parameters are available in the product specifications. We analyze different detection configurations and optical components are compared to find the optimal ones. The results are discussed in Sec. 3.1 with four examplary design cases. Note that, the SNR are approximated due to a few assumptions to derive the equations, autofluorescence and alignment factors.

## 3. Results and discussions

### 3.1 Modeling and evaluating influential factors

**Fig. 4.**
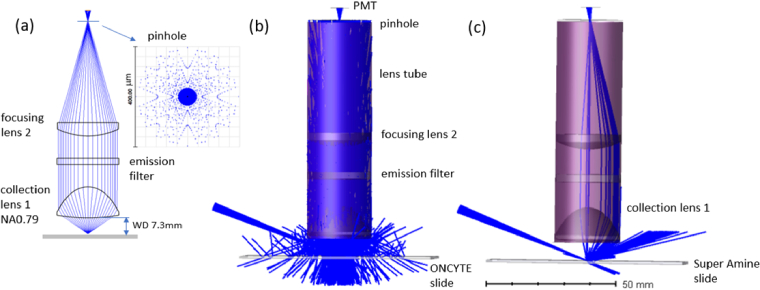
(a) collection optical path using two off-the-shelf aspheric lenses, with a high NA of 0.79, long WD of 7.3 mm. Scatter modeling of the CFR module with tilt 60-degree dark field illumination using (b) Lambertian scattering model for ONCYTE and (c) ABg scattering model for Super Amine.

To design the collection optics with high NA, long WD and low cost, we select two off-the-shelf aspheric lenses (Thorlabs ACL25416-A, AL2550-A) as the collection lens and focusing lens, ensuring a point-by-point scan with a high NA of 0.79 and long working distance of 7.3 mm. Optical ray tracing shows a focused spot size to go through the pinhole (Thorlabs P200D, ϕ200µm), as seen in Fig. 4(a). With the magnification of 3.12, this leads to a conjugated pinhole size of ∼65µm on the sample slide.

On top of a high throughput to efficiently collect signals, the CFR module should consider a high SNR to avoid false positive readings. Using the proposed SNR evaluation method in Sec. 2.4, we investigate three influential factors, including the sample slides, perpendicular/tilt illumination and filters, for improving the signal intensity and reducing the noise. Most fluorescence microscope designs have ignored the scattered laser light, as either the sample volume is sufficient, or the fluorophore concentration is high. In such a sub-micro-liter, low-abundant fluorescence detection, the laser light scattered back to the emission path can be a considerable source of noise.

For modelling the scatter noise, two representative sample slides are selected, ONCYTE (Grace bio-labs, 705278) and Super Amine (Arrayit, SMM2). By measuring the bilateral scatter distributed function (BSDF) data with the scatterometer (REFLET bench) and then fit the data using the ABg surface scattering model [29], we have performed non-sequential ray tracing with 1 million rays using Zemax OpticsStudio [30]. Figure 4(b) shows the scattering modelling of the ONCYTE and Super Amine slides with 60-degree illumination, where the title angle is with respect to the sample surface normal vector. Note that the ONCYTE slide can induce much larger laser noise due to the high Lambertian scattering on the slide surface. The scattering is quantified as a percentage to further determine the laser light that can reach the detector, and the detailed implementation has been illustrated in [29].

**Fig. 5.**
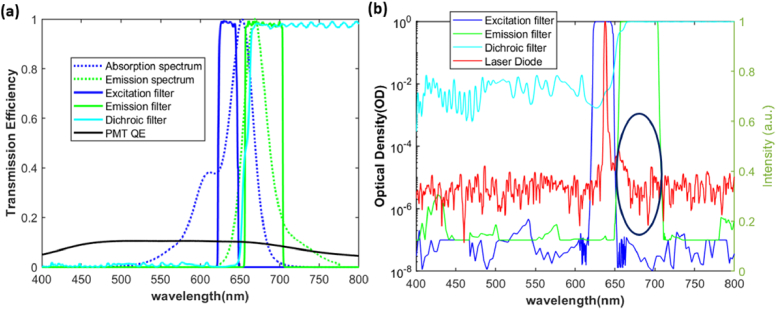
(a) The corresponding spectra of fluorophore Alex Fluor 647, filter set and PMT quantum efficiency (QE) for SNR calculation, (b) The spectra in logarithmic coordinates to clearly see the potential side wavelengths (highlighted by a black circle) that can pass through the emission and dichroic filter while blocking by the emission filter.

To reject the laser beam, a set of excitation, emission and dichroic filters (Semrock LED-Cy5-A), and a very compact PMT detector (Hamamatsu µPMT H10721-01) are selected. All the involved components for SNR calculation are summarized in Table 1. The laser diode spectrum is measured using a commercial spectrometer (Avantes AvaSpec-2048). The spectra of the filter set and the PMT quantum efficiency (QE) are provided by the supplier plotted in Fig. 5(a), estimated from a batch of standard products, but each individual filter might have a deviation from the given spectra.

**Table 1.** Collected parameters of the setup components

	**Type No.**	**Parameters**	**Collection method**
**Laser**	Thorlabs CPS635F	Typical power 5mW	Measured spectrum
**Lenses**	Thorlabs ACL25416-A, AL2550-A	NA = 0.79, T_opt1_ = 0.9, T_opt2_ = 0.9	Nominal values from Thorlabs
**Filters**	Semrock LED-Cy5-A	FF01-635/18, FF01-680/42, FF652-Di01 spectra	Nominal spectra from Semrock
**Sample**	ThermoFisher Alexa Fluor 647 in distilled water	DMAC = 270000, quantum yield = 0.33, normalized absorption and emission spectra	Nominal parameters from ThermoFisher
**Slide**	Super Amine (Arrayit, SMM2)	Scattered laser yield	Simulation in Zemax
**Pinhole**	Thorlabs P200D	Pinhole diameter = ϕ200µm	Nominal parameters from Thorlabs
**Detector**	Hamamatsu µPMT H-12400	Quantum efficiency = 10.3% (630 nm), radiant sensitivity = 1.5 × 10^4^A/W, average dark current = 0.6 nA (max. 6 nA)	Nominal parameters from Hamamatsu

The SNR of four exemplary configurations is calculated using different illuminations and filter combinations. The modelling sample concentration is 2.5 ng/ml, 40nl. With all the parameters available following the flowchart of Fig. 3, we obtained the generated noises and signals, as seen in Table 2. The first interesting finding is that, even though the laser has a very narrow FWHM (0.94 nm), the extremely weak power from non-lasing wavelengths can still flood the collected fluorescence signals if no excitation filter is used. To find out the reason, the spectra of the filters and the measured laser spectrum are plotted in logarithmic coordinates, as seen in Fig. 5(b). We notice that the unblocked laser noise can go into the collection path as highlighted in the black circle, if only the emission filter is used. Secondly, the dark current noise is typically the most influential factor in supersensitive fluorescence detection, but laser scatter noise can also have considerable impact if the filters are not properly chosen, as seen in the SNR results of Figs. 2 and 4.

**Table 2.** SNR simulation with different confocal fluorescence configurations at 2.5 ng/ml Alexa Fluor 647 dissolved in distilled water

Power: mW	Bright field illumination (0 deg)		Dark field illumination (60 deg)	
Configuration	1	2	3	4
Filters	FF01-635/18, FF01-680/42, FF652-Di01	FF01-680/42, FF652-Di01	FF01-635/18, FF01-680/42	FF01-680/42
Laser scatter noise	7.42e-12	2.79e-07	8.2e-12	1.33e-08
Dark noise (average)	4e-11			
Signal	1.16e-08	1.17e-08	1.29e-08	1.31e-08
Estimated SNR	**244.90**	0.04	**268.34**	0.98

With a proper selection of the filter set and collection optics, the slanted illumination (Fig. 3) has the best SNR, slightly better than the perpendicular illumination (Fig. 1). Note that Fig. 3 also has one filter less, resulting in a slightly higher throughput and a lower cost. More combinations of different filters are easily available, e.g., using BLP01-635R instead of FF01-680/42. As Fig. 1 tends to have less alignment steps for practicability, which excludes the potential large errors from misalignment, this classic optical path is chosen for the proof-of-concept demonstrator.

### 3.2 Implementation of setup and experiments

**Fig. 6.**
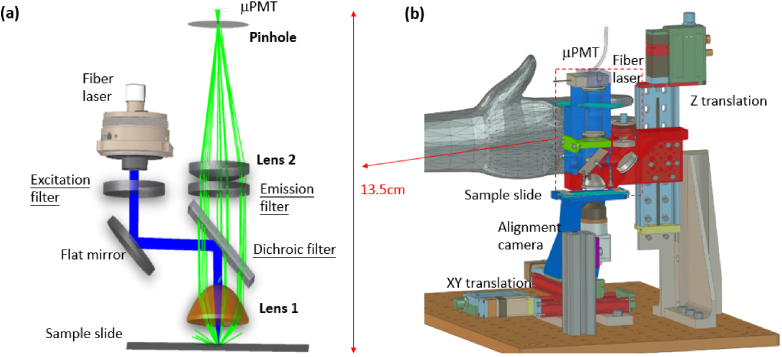
The compact CFR experimental setup (a) its optical path consists of the excitation laser beam denoted by blue rays and the emitted fluorescent signals in green rays and (b) the overall mechanical design including the alignment camera, the xyz translation stages for scanning and fine adjustment. A sample slide is inserted in the slot, designed the same as in the DSS dispensing module.

With the SNR modelling results in Table 2, we choose the Fig. 1 with all the selective optical components and build the setup as seen in Fig. 6. An integrated imaging camera (IDS U3-3060CP-C-HQ) underneath the sample slide holder is used in assisting the coaxial alignment of the excitation laser and the collection optics.

The fluorescent sample is prepared by using a secondary antibody conjugated with a fluorophore, the Alexa Fluor Plus 647 (life technologies, cat. no. A32733), abbreviated as Ab-fluor. The sample was purchased as a solution with an initial concentration of 1 mg/ml and diluted in dH_2_O to a final concentration of 2.5 ng/ml. During the DSS process, around 1 µl of this sample solution was loaded in the micro-printing pin and then dispensed on a standard 1 mm thick Super Amine glass slide. A tungsten heater was powered by rectangular waveform pulses to jet tiny droplets and generate spots of accumulated sample with diameters ∼200 µm per spot. The full implementation of the DSS dispenser has been described in our previous work [22]. Each sample spot had 40 dispensed jets with around 1 nano-liter per jet. The sample slides with recorded positions of each spot are then transferred from the DSS dispensing to the CFR module for fluorescence measurements.

**Fig. 7.**
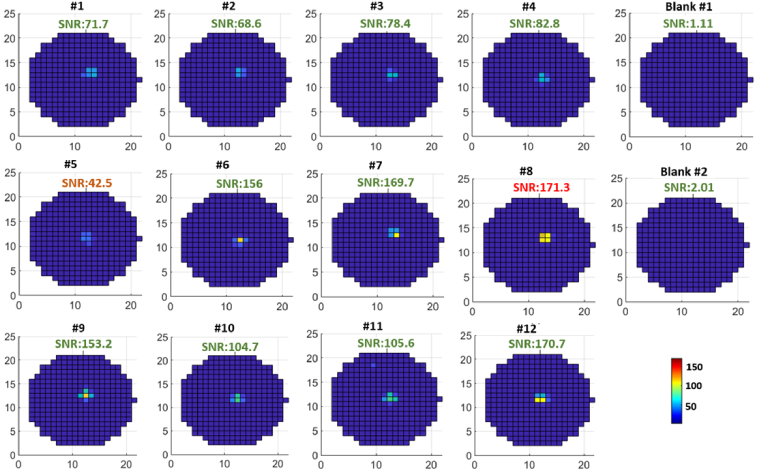
The signal to noise ratio (SNR) maps of the scanned 12 sample spots (secondary antibody conjugated with Alexa Fluor 647 and diluted to 2.5 ng/ml in distilled water) and the 2 blank control groups (pure distilled water) using the developed CFR module. The maximum SNR is displayed on top of each measurement, ranging from 42.5 to 171.3 for the samples, and SNR < 3 for the blank control groups.

During the CFR scanning measurement, the alignment camera is first used to locate the spot positions on the sample slide under a blue LED illumination. It was seen that there was an offset of 100-200 µm in both x- and y- direction between the CFR and DSS module coordinates. It could be minimized by optimizing the system calibration of both modules. For the moment, the CFR stage scans at a grid spacing of 75 µm with a circular diameter of 1.5 mm, which can mitigate the small offset errors. The amplitude voltage of the µPMT detector is set at 1.1 V. Each sample spot accounts for ∼350 readout values with a circular region, so the measurement time is 0.18 s/spot with a sampling rate of 2000Hz. The scanning results of 12 samples plus 2 blank control spots are shown in Fig. 7. Since the effective signals typically account for a few adjacent readouts, we filter out the single “hotspot” readout as noise. The background noise for each sample spot is determined by the average of the minimum 330 readings. The maximum SNR is then calculated as the ratio of the maximum signal to the background noise and specified on each sample. Clearly, the 2.5 ng/ml concentration can be detected with SNRs in the range of [42.5, 171.3], while the two blank sample spots have a maximum SNR of 2.01.

**Fig. 8.**
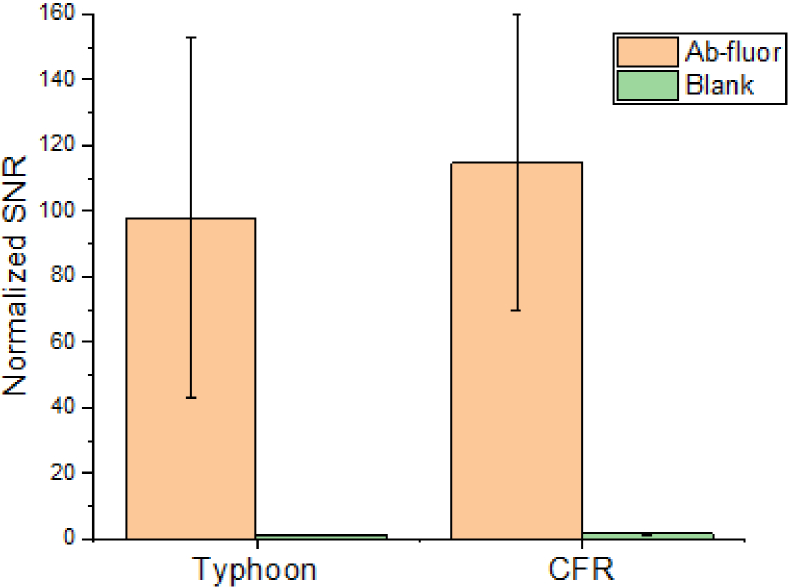
Performance comparison of the Typhoon scanner and the developed CFR module in terms of normalized SNR and standard deviation. The average SNR is 114.6 for CFR module, and 97.8 for Typhoon scanner, with comparable standard deviation.

For comparison, we also measured the same 14 sample spots using a commercial high-end fluorescence scanner (GE Typhoon imager). The SNR is normalized by making the blank group SNR as 1. The original data is shown in Supplement 1, with the normalized SNR in the range of [37, 215.9]. Figure 8 shows the normalized SNR and the standard deviation level for both devices. Note that, the results of CFR have filtered out the single “hotspot” values. In terms of the measured average normalized SNR, our CFR scanner gives a value of 114.6, while for the Typhoon we find 97.8 with a much finer scanning step of 10µm. With respect to the standard deviation level, they are also comparable. Both devices show the maximum SNR lower than the simulation SNR in Table 2, probably due to the autofluorescence of other components which is ignored in the simulations or due to fluctuant readout noises from the background.

**Fig. 9.**
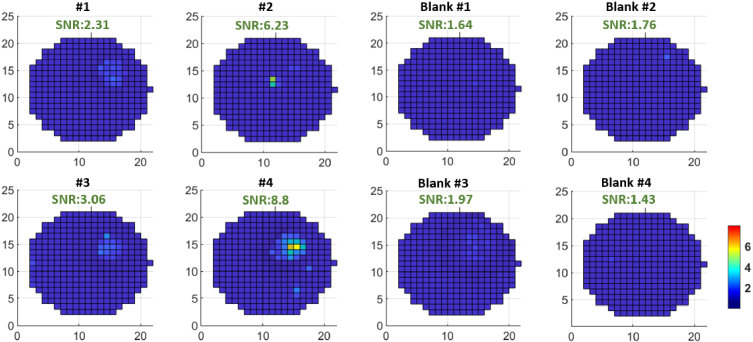
The signal to noise ratio (SNR) maps of the scanned sample spots (secondary antibody conjugated with Alexa Fluor 647 and diluted to 0.8pg/ml in artificial urine) and the blank control groups (blank #1 and #3 are the spots with pure artificial urine and blank #2 and #4 with pure distilled water) using the developed CFR module. The maximum SNR is displayed on top of each measurement. 3 of 4 sample spots show SNR > 3, and all four blank control groups show SNR < 3.

We made a measurement with the developed CFR module of artificial urine for highly challenging low-abundant samples. Simulations using the developed SNR calculation program show that the SNR is barely detected (∼3.78) when the low-abundant sample concentration equals 0.8 pg/ml and if the pyroelectric dispenser can accumulate the droplets with a diameter of ∼80 µm per spot. The same settings as above have been used for the CFR and DSS modules for the jetting process and the sample measurement. The blank control spots are divided into two groups, with blank #1 and #3 being the pure artificial urine and blank #2 and #4 being pure distilled water. As seen in Fig. 9, measurements show that 3 out of 4 samples have an SNR over 3, while all the blank spots are less than 3 with a maximum SNR of 1.97. The measurements of the same samples using a commercial fluorescence scanner InnoScan 710 are shown in Supplement 1. With such low-abundant samples, the geometry and uniformity of each spot is complicated and difficult to give an identical diameter. Thus, the SNR calculation varies a lot based on the selected region of interest, making it very difficult to generate a fair comparison.

## 4. Conclusions

We propose a hybrid method of using pyroelectric dispensing and confocal fluorescent detection together for sub-attomole analyte, promising for point-of-care diagnosis. The pyro-electric droplet-split-and-stack (DSS) dispensing can accumulate the sample analytes into a stacked micro-region, meanwhile recording the spot positions. Without the necessity to clearly “see” the spot, the high-numerical aperture (NA = 0.79), long working distance (WD = 7.3 mm) confocal fluorescence readout (CFR) module uses merely two off-the-shelf optical lenses, resulting in a compact setup and high performance in terms of SNR for low abundant analytes. Furthermore, the CFR can scan a smaller region around the targeted sample spots, instead of scanning the whole slide as in most high-end confocal fluorescence scanners. In terms of time cost, the scanning speed of the proposed CFR is about 0.18 s/spot, so it takes only 2.52 seconds to obtain the results of Fig. 7. As a comparison, the commercial InnoScan 710 usually takes 3-5 minutes per slide, while the Typhoon image scanner has a similar time cost.

We also present a modeling method that considers the impact of the influential noises and the generated fluorescence signal to determine the SNR for different confocal fluorescence detection setups. The developed simulation tool is applicable for various confocal configurations (e.g., different illumination angles and filters), thus it is feasible to form a quick comparison among different options. It can provide guidance to identify the dominant noise for further improving the detection limit, prior to building a prototype that is time-consuming, expensive, sometimes even with over-functional components (e.g., multiple filter set). Both simulation and experimental results show the success detection of low volume, low abundance Ab-fluor (0.04µl, 0.67 attomoles) with an average SNR ∼100, which is comparable to a high-end Typhoon imager. Note that, the experimental results might have deviations from simulation due to many other factors and assumptions made in the modeling, such as autofluorescence, sample preparation and alignment errors. To step further, our developed prototype has showed promising results for a very challenging low-abundance secondary antibody sample conjugated with Alexa Fluor 647 in artificial urine (0.04µl, 0.21 zeptomoles).

## Data Availability

The authors confirm that the data supporting the findings of this study are either available within the article and its supplementary materials or could be obtained from the authors upon reasonable request.

## Supplemental document

See Supplement 1 for supporting content.
