# Supplementary material for: Miniaturized, high numerical aperture confocal fluorescence detection enhanced with pyroelectric droplet accumulation for sub-attomole analyte diagnosis
Source: Biomed Opt Express. 2023 Nov 3;14(12):6138–50. doi: 10.1364/BOE.504757 (PMC10898570; doi:10.1364/BOE.504757)
Supplement: Supplementary file 1 [file boe-14-12-6138-s001.pdf]

Supplemental Document

Biomedical Optics EXPRESS

Miniaturized, high numerical aperture confocal fluorescence detection enhanced with pyroelectric droplet accumulation for sub-attomole analyte diagnosis: supplement

YUNFENG NIE,[1,*] UUSITALO SANNA,[2] TEEMU SIPOLA,[2] ANNUKKA KOKKONEN,[2] INKA PÄKKILÄ,[2] JUHA SUMEN,[2] KATARIINA RAHKAMAA-TOLONEN,[2] VOLODYMYR TKACHENKO,[3] VERONICA VESPINI,[3] SARA COPPOLA,[3] PIETRO FERRARO,[3] SIMONETTA GRILLI,[3] AND HEIDI OTTEVAERE[1]

[1] *Vrije Universiteit Brussel and Flanders Make, Brussel Photonics, Dept. of Applied Physics and Photonics, Pleinlaan 2, 1050 Brussels, Belgium*
[2] *VTT Technical Research Centre of Finland Ltd, Kaitoväylä 1, FI-90571 Oulu, Finland*
[3] *Institute of Applied Sciences and Intelligent Systems, National Council of Research (CNR-ISASI), Via Campi Flegrei 34, 80078 Pozzuoli, Italy*
[*] *Yunfeng.Nie@vub.be*

This supplement published with Optica Publishing Group on 3 November 2023 by The Authors under the terms of the Creative Commons Attribution 4.0 License in the format provided by the authors and unedited. Further distribution of this work must maintain attribution to the author(s) and the published article's title, journal citation, and DOI.

Supplement DOI: https://doi.org/10.6084/m9.figshare.24459235

Parent Article DOI: https://doi.org/10.1364/BOE.504757

Supplementary materials for

Miniaturized, high numerical aperture confocal fluorescence detection enhanced with pyroelectric droplet accumulation for sub-attomole analyte diagnosis

Yunfeng Nie,[1,*] Uusitalo Sanna[2], Teemu Sipola[2], Annukka Kokkonen[2], Inka Päkkilä[2], Juha Sumen[2], Katariina Rahkamaa-Tolonen[2], Volodymyr Tkachenko[3], Veronica Vespini[3], Sara Coppola[3], Pietro Ferraro[3], Simonetta Grilli[3] and Heidi Ottevaere[1]

[1]Vrije Universiteit Brussel and Flanders Make, Brussel Photonics, Dept. of Applied Physics and Photonics, Pleinlaan 2, 1050 Brussels, Belgium
[2]VTT Technical Research Centre of Finland Ltd, Kaitoväylä 1, FI-90571 Oulu, Finland
[3]Institute of Applied Sciences and Intelligent Systems, National Council of Research (CNR-ISASI), Via Campi Flegrei 34, 80078 Pozzuoli, Italy
[*] Yunfeng.Nie@vub.be

S1. Beer-Lambert Law to calculate absorbed laser power

To evaluate the SNR performance of various confocal fluorescence detection configurations, we need to quantify both the generated fluorescence signal and all types of noise on the photodetector.

In terms of laser illumination, the laser power P_0 reaching the sample is calculated by

$$P_0(\lambda) = P_L.\Phi_L(\lambda).T_{ex}(\lambda).T_{opt1}(\lambda).R_{Di}(\lambda) \quad \text{(S1)}$$

Where, P_L is the total laser power (W), Φ_L is normalized laser spectrum, T_{ex}, T_{opt1} are the transmission spectra of the excitation filter and other optics in the excitation light path. R_{Di} is the reflection spectrum of the dichroic filter; if no dichroic filter, $R_{Di}=1$.

The laser induced fluorescence signal is proportional to the total absorbed laser power. Based on Beer-Lambert law [1], the absorbed power by the sample is

$$P_{abs}(\lambda) = P_0(\lambda) - P_d(\lambda) = P_0(\lambda)\left(1 - e^{-\varepsilon(\lambda).c.d}\right) \quad \text{(S2)}$$

Where, $P_0(\lambda)$ and $P_d(\lambda)$ are the incident and outgoing laser radiant flux (Watt), c is molar concentration (M) and $\varepsilon(\lambda)$ is extinction coefficient ($M^{-1}cm^{-1}$), d is the sample thickness.

Provided a low concentration, the absorbed light power is approximately obtained by

$$P_{abs}(\lambda) \approx P_0(\lambda).\varepsilon(\lambda).c.d \quad \text{(S3)}$$

Note that, the linear approximation is not valid at high dye concentration due to quenching. The extinction coefficient can be also denoted by [2]

$$\varepsilon(\lambda) = \log(10).DMAC.\Phi_A(\lambda) \tag{S4}$$

Where, DMAC stands for the maximum decadic molar absorption coefficient and is a standard parameter for fluorophore dyes, Φ_A is the normalized fluorophore absorption spectrum. The total absorbed power is equal to

$$\overline{P}_{abs} = \eta_L.\eta_N \int P_{abs}(\lambda) d\lambda = \log(10).\mathrm{DMAC}.c.d \int P_0(\lambda)\Phi_A(\lambda) d\lambda \tag{S5}$$

Here, the two critical ratios: η_L is the absorbed laser percentage by the fluorophores and η_N is the effective fluorophore percentage per laser scan, as explained in Fig. 3 of the main text.

S2. Measurement results using Typhoon image scanner

We measured the sample spots (secondary antibody conjugated with Alexa Fluor 647 and diluted to 2.5 ng/ml in distilled water) using a commercial high-end GE Typhoon fluorescence scanner. The Typhoon scanner has a high resolution with 10um per step, but it cannot automatically find the positions of the sample spots. The SNR results are obtained by making the blank group SNR as 1. We manually select each sample spot and draw a circle with a diameter of 200µm as the region of interest for each SNR measurement as seen in Fig. S1.

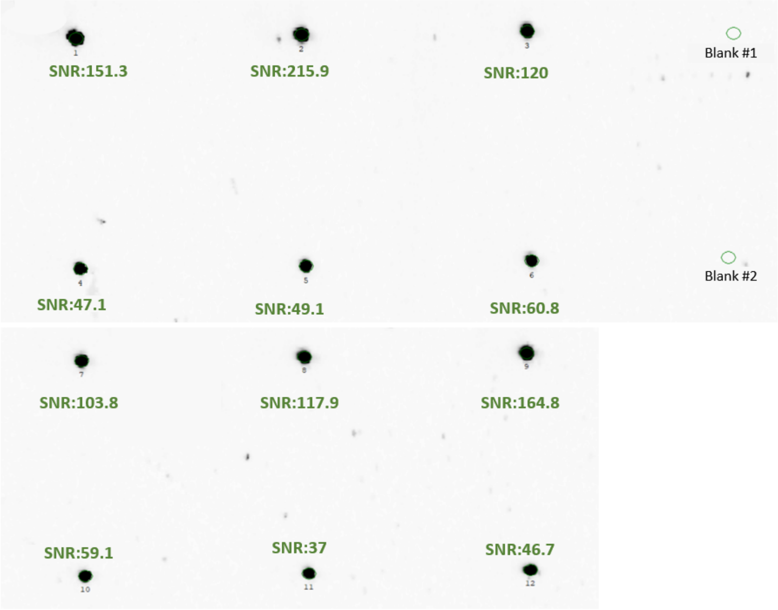

Figure S1. The signal-to-noise-ratio maps of the scanned sample spots (secondary antibody conjugated with Alexa Fluor 647 and diluted to 2.5 ng/ml in distilled water) and the blank control groups (pure distilled water) using a high-end commercial GE Typhoon fluorescence scanner. The SNR of each sample spot is calculated by manually drawing a region of 200µm diameter. Notice that the scanner cannot locate the spot, so the full slide is scanned where some “hotspot” values can be seen clearly.

S3. Measurement results using InnoScan 710

We measured these sample spots (secondary antibody conjugated with Alexa Fluor 647 and diluted to 0.8pg/ml in artificial urine) using a commercial fluorescence scanner InnoScan 710 as seen in Fig. S2. The InnoScan has a high resolution with 3um per step, but it also needs to scan the full slide to get the signals out. As the SNR of each sample spot varies largely with the region of interest, it is difficult to determine the SNR of each spot.

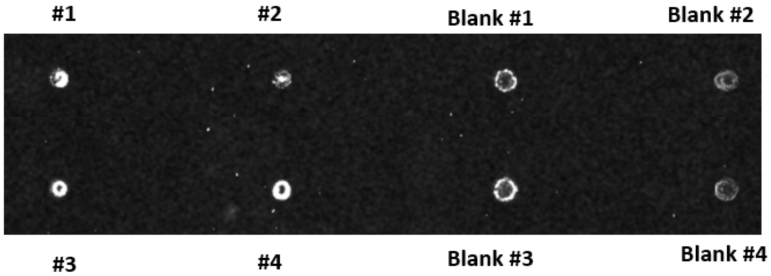

Figure S2. The signal-to-noise-ratio maps of the scanned sample spots (secondary antibody conjugated with Alexa Fluor 647 and diluted to 0.8pg/ml in artificial urine) and the blank control groups (blank #1 and #3 are the spots with pure artificial urine and blank #2 and #4 with pure distilled water) using a commercial fluorescence scanner InnoScan 710.

References

1. Lakwicz, J. R. *Principles of Fluorescence Spectroscopy*. (Springer, 2006).
2. Anderson, N., Prabhat, P. & Erdogan, T. Spectral modelling in Fluorescence microscopy. Preprint at http://www.semrock.com/Data/Sites/1/semrockpdfs/spectral_modeling_in_fluorescence_microscopy.pdf
